# Mesenchymal stem cell therapy for acute radiation syndrome: innovative medical approaches in military medicine

**DOI:** 10.1186/s40779-014-0027-9

**Published:** 2015-01-30

**Authors:** Erik B Eaton, Timothy R Varney

**Affiliations:** United States Army Medical Research Institute of Chemical Defense, 3100 Ricketts Point Road, Aberdeen Proving Ground, Maryland, 21010 US

**Keywords:** Acute radiation syndrome, Mesenchymal stem cell, Cell therapy, Hematopoietic syndrome, Gastrointestinal syndrome, Radiation injury

## Abstract

After a radiological or nuclear event, acute radiation syndrome (ARS) will present complex medical challenges that could involve the treatment of hundreds to thousands of patients. Current medical doctrine is based on limited clinical data and remains inadequate. Efforts to develop medical innovations that address ARS complications are unlikely to be generated by industry because of market uncertainties specific to this type of injury. A prospective strategy could be the integration of cellular therapy to meet the medical demands of ARS. The most clinically advanced cellular therapy to date is the administration of mesenchymal stem cells (MSCs). Results of currently published investigations describing MSC safety and efficacy in a variety of injury and disease models demonstrate the unique qualities of this reparative cell population in adapting to the specific requirements of the damaged tissue in which the cells integrate. This report puts forward a rationale for the further evaluation of MSC therapy to address the current unmet medical needs of ARS. We propose that the exploration of this novel therapy for the treatment of the multivariate complications of ARS could be of invaluable benefit to military medicine.

## Introduction

The inclusion of cellular therapies in the treatment of battlefield injuries provides a novel and promising approach for addressing long-standing challenges in tissue repair with regard to both structural and functional improvements. The results of currently published investigations describing adult stem cell efficacy in a variety of injury and disease models demonstrate the unique qualities of reparative cell populations present in adult tissues. Bone marrow-derived mesenchymal stem cells (MSCs), adipose derived stem cells, and endothelial progenitor cells, for example, exhibit a remarkable capacity to adapt to the requirements of the damaged tissue in which the cells integrate. In this study, we focus on the potential use of MSC therapy as a medical countermeasure for the treatment of acute radiation syndrome (ARS). MSC therapy represents a single medical intervention that can simultaneously provide a broad range of therapeutic efficacy, with local activity, at multiple tissue and organ sites. Although ARS is rare, it is a complex and medically challenging disorder that has the potential for large-scale incidence on the battlefield or in conjunction with a domestic terrorist attack. Currently, medical intervention for numerous aspects of ARS is limited to supportive care.

Since the end of the Second World War, nearly all radiation injuries have been caused by accidents in the medical and nuclear power industries. However, the rise in global terrorism, the proliferation of nuclear technology, and the undocumented dispersal of enriched uranium, plutonium, and other radioactive compounds have intensified concerns regarding the use of a radiological or nuclear weapon to inflict extensive military casualties, civilian casualties, or both. Detonating an improvised radiological device or nuclear warhead could necessitate the medical management of hundreds to thousands of patients with ARS, which typically occurs to some degree following a whole body dose of 1 Gray (Gy) or more [1,2].

The underlying pathology of ARS involves physical and chemical damage to DNA, which affects the rapidly dividing cells of the hematopoietic system and the gastrointestinal (GI) tract. As a result, ARS symptoms are often subclassified into the hematopoietic and GI syndromes, which occur simultaneously at higher exposure levels. As discussed below, the therapeutic benefit of MSC therapy for these individuals could include the facilitation of hematopoietic recovery, enhancement of healing of the GI tract and the skin, and the possible mitigation or treatment of a variety of additional ARS complications.

### MSC biology and clinical use

MSC-based therapeutics have emerged as the most clinically advanced multipotent stem cell therapy to date. At least 178 Phase II or Phase III trials have been completed or are currently recruiting patients to evaluate MSC therapy for the treatment of a diverse array of indications (www.clinicaltrials.gov, September 2014 [3]). A commercial product consisting of purified MSCs has received regulatory approval in both Canada and New Zealand for the treatment of graft vs. host disease (GvHD), a potentially lethal complication of allogeneic bone marrow transplant arising from the severe immune reaction of the donated immune cells against the host tissues (Osiris Therapeutics, Inc., Columbia, MD). The currently marketed cell-based treatment regimen does not contain embryonic cells or any constituent derived from fetal sources. Although other forms of stem cell therapies based on induced pluripotent stem cells, embryonic stem cells, or umbilical cord blood continue to show promise in pre-clinical studies, MSC therapy represents an approach that is rapidly gaining acceptance in clinical practice. No other multipotent stem cell therapy has established an extensive safety profile in the clinical setting.

Intravenously (IV) infused MSCs have been shown to specifically migrate to sites of tissue damage in multiple preclinical injury models [4,5]. MSC infusion mimics a naturally occurring process in which endogenous MSCs leave the bone marrow compartment in response to injury, enter the circulation, and travel to sites of tissue damage, guided by chemotactic homing signals released at each compromised site. The clinical development of MSC formulations for therapeutic use has involved the isolation of MSCs from bone marrow and expansion in culture [6]. Numerous studies have indicated that donor-derived, IV-administered MSCs retain the ability to localize to damaged tissue and facilitate repair in a variety of injury and disease settings. Cell culture-expanded MSCs demonstrate the potential to form several specialized cell types, including neurons, skin, bone, fat, cartilage, tendon, muscle (cardiac and skeletal), epithelium (lung, gut, and kidney), and many others. Once engrafted within damaged tissues, MSCs participate in the healing process both directly, through differentiation to replace lost cell types, and indirectly, through the local secretion of cytokines and other bioactive molecules that facilitate a reduction in inflammation, the inhibition of scar formation, and the enhancement of endogenous mechanisms of tissue reconstruction [7].

The molecular basis for MSC homing to injury sites has been evaluated by several independent laboratories using both *in vitro* and *in vivo* approaches. The data describe MSC chemotaxis toward a variety of chemokines, including monocyte chemoattractant protein-1 (MCP-1), macrophage inflammatory protein-1α (MIP-1α), interleukin-8 (IL-8), and stromal derived factor-1 (SDF-1) [8,9]. For example, Wang et al. [8] observed *in vitro* MSC migration toward purified MCP-1, MIP-1α, and IL-8, as well as toward extracts prepared from brain tissue injured by oxygen deprivation (ischemic injury). The involvement of the specific chemokine in question was verified by the inhibition of directional migration in the presence of a blocking antibody to each cytokine.

Once engrafted to damaged tissues, MSCs elicit a broad range of effects with regard to modulation of the inflammatory response to injury. MSCs express a low level of major histocompatibility (MHC) class I molecules, but they lack expression of MHC class II and the B7 co-stimulatory molecule. These molecules are involved in antigen presentation at the cell surface. MSCs are therefore rendered “immune privileged” by the capacity to evade recognition by both CD4^+^ T helper and CD8^+^ cytotoxic T cells. In addition to being immune privileged MSCs also actively attenuate immune activity. Cell surface markers of lymphocyte activation, including CD25, CD38, and CD69, have been shown to decrease in the presence of MSCs. T cell proliferation is inhibited by MSCs through a block in cyclin D2 expression, resulting in cell cycle arrest. Finally, MSCs have also been shown to inhibit the innate immune response by blocking the IL-2-mediated activation of natural killer cells [10].

In addition to the initial anti-inflammatory properties of MSCs, engraftment induces the local secretion of multiple paracrine factors that facilitate wound healing. These factors include angiogenic, anti-apoptotic, mitogenic, and homing signals, such as vascular endothelial growth factor (VEGF), hepatocyte growth factor (HGF), insulin-like growth factor-1 (IGF-1), basic fibroblast growth factor (bFGF), and SDF-1, resulting in the accumulation of several distinct populations of blood vessel precursor cells and other tissue-specific progenitor cells [7,9,11,12].

Both preclinical and clinical studies have demonstrated the safety and efficacy of allogeneic (non-immunologically matched) MSC delivery (see recent reviews by Inamdar et al. [13]; Sheng et al. [14]; and Honmou et al. [15]). The general consensus for efficacy with the use of allogeneically derived MSCs is increasingly ascribed to anti-inflammatory and paracrine activity [16]. However, significant interest still remains in evaluating the role of MSCs, as a stem cell population, in promoting wound repair by direct differentiation to replace cells lost to injury. In the context of addressing the consequences of battlefield injury, we see a distinct and specific value for the use of either allogeneic or autologous (patient-derived) MSC therapy. The rationale for determining the MSC source will be dependent on the treatment goals for each indication. For example, early treatment for a blast injury may be focused on the anti-inflammatory and paracrine properties of MSCs. Stockpiled, “off-the-shelf” allogeneic MSCs would be appropriate for use in this case and could be administered in a Combat Support Hospital. The use of MSCs for reconstructive procedures to replace significant tissue loss, however, would likely require autologously derived cells that would not be cleared by the immune system after the cells mature and begin to express donor-specific proteins.

The characteristic anti-inflammatory, tissue repair, and hematopoietic support capabilities of this particular stem cell population suggest that MSC therapy may be ideal for inclusion in medical preparedness planning for a radiological or nuclear event. Given the broad applicability of MSC treatments to numerous diverse injuries and diseases, the development of MSC treatment for ARS is also likely to benefit treatment strategies for a wide array of additional conditions.

### Therapeutic potential of MSC treatment of hematopoietic syndrome

The hematopoietic syndrome is the principal cause of mortality in radiation exposure doses from 1 to 10 Gy [1]. The manifest illness is typically characterized by intense immunosuppression, anemia, and thrombocytopenia resulting from the death and/or reduced replication of hematopoietic progenitor cells following irradiation. Current data from studies on animals and humans suggest that the mean lethal dose of whole body radiation required to kill 50% of patients in 60 days is between 3 and 4 Gy. When patients are given appropriate medical management with intravenous hydration, antiemetics, analgesics, antibiotics, and blood transfusions, the LD_50_ is estimated to lie between approximately 6 and 7 Gy [1]. Current guidelines recommend short-term cytokine therapy with granulocyte colony-stimulating factor (Neupogen® [Filgrastim, Amgen, Inc.]), granulocyte-macrophage colony-stimulating factor (GM-CSF), and pegylated G-CSF to facilitate hematopoietic recovery [17].

Both *in vitro* and *in vivo* (including clinical) data indicate that the addition of MSC therapy to standard care can promote more rapid recovery of the hematopoietic system. MSCs constitutively secrete a broad range of hematopoietic cytokines, such as IL-6, IL-7, IL-8, IL-11, IL-14, IL-15, macrophage colony-stimulating factor, Flt-3 ligand, and stem-cell factor (SFC). IL-1a induces MSC production of IL-1a, leukemia-inhibiting factor, G-CSF, and GM-CSF [18]. *In vitro* evaluations have provided detailed evidence that these factors, as well as other members of the MSC secretome (the array of proteins produced and secreted by MSCs), support the growth of hematopoietic stem cells (HSCs) and their progeny in an *ex vivo* environment. MSC deposition of extracellular matrix constituents that are native to the bone marrow compartment is likely to significantly contribute to this phenomenon. Relevant to hematopoietic recovery after radiation exposure, *in vitro* studies have also shown a positive influence of the presence of MSCs on the expansion of irradiated blood cell precursors: irradiated CD34^+^ cells demonstrate a several-fold growth increase when cultured in the presence vs. the absence of MSCs [19].

At higher radiation exposure levels, patients will require an HSC transplant. In a mass casualty scenario, the timely treatment of large numbers of patients will be challenging and will require innovative medical intervention. Preclinical work in animal models has evaluated the influence of HSC–MSC co-transplant on hematopoietic recovery. The results of these analyses suggest that HSC–MSC coinfusion facilitates HSC engraftment and hematopoietic recovery [20,21]. Furthermore, MSC coinfusion increases the success rates of HSC transplants, even under sub-optimal transplant conditions. These scenarios include the transplantation of a limited number of HSCs [21,22] or situations in which transplanted HSCs have been obtained from two immunologically disparate donors [23].

Fortunately, the promising results obtained in preclinical studies are being realized in the clinical setting as well. A successful clinical use has been the coinfusion of HSC–MSC following ablative radiation or chemotherapy for the treatment of both malignant and non-malignant disorders. A large body of data supports the concept that MSC therapy provides a safe and effective treatment modality to facilitate hematopoietic recovery and overall survival following medical intervention that involves an HSC transplant (reviewed in Tolar [24]). The majority of published reports discuss clinical outcomes from small or single-patient case studies. Despite differences in the particular medical disorder, patient demographics, and treatment regimen, the collective results suggest an increase in both HSC graft stability and overall patient survival.

Importantly, larger clinical trials have evaluated results from a treated subject pool against relevant historical control patients. These studies have demonstrated significant efficacy with the use of HSC–MSC coinfusion to promote the rapid recovery of cellular blood constituents. For example, a Phase I/II clinical study by Ball et al. ([25]) followed 14 patients who were co-transplanted with donor MSCs and human leukocyte antigen (HLA)-disparate peripheral blood HSCs. The results were compared to 47 historic control subjects. Study subjects were treated at the same two medical facilities as control patients. The infusion dose ranged from 1 to 5 million MSCs/kg body weight. MSC-treated subjects experienced faster recovery in total leukocyte count (above 1.0 × 10^9^/L) in comparison to historic controls, with patients in this study having a mean time to recovery of 11.5 days and the control group recovering in 14.9 days (*P* = 0.009). Although control subjects experienced a graft failure rate of 15%, all patients who were given MSCs in this study showed sustained hematopoietic engraftment without adverse reactions.

Rapid hematopoietic reconstitution was also reported for 28 subjects enrolled in a breast cancer study that had received high-dose chemotherapy followed by the coinfusion of peripheral blood stem cells and MSCs. In this investigation, all patients received 1 to 2.2 million autologous MSCs/kg body mass in conjunction with G-CSF therapy. Neutrophil recovery (judged as greater than 500/μl) ranged from 6 to 11 days, with an average of 8 days. Platelet engraftment (judged as greater than 20,000/μl) ranged from 4 to 19 days, with an average of 8.5 days [26]. Historical control data specifically relevant to this institute or by these researchers are not reported. However, these values for neutrophil and platelet recovery are more favorable compared with the results of large clinical trials published elsewhere in which patients received G-CSF treatment (e.g., Amadori [27], Usuki [28], Nivison-Smith [29], Godwin [30], Lowenberg [31], Hassan [32], Rowe [33], Stone [34], Ottmann [35]).

Depending on the scale of a radiological or nuclear incident, medical response logistics, and the availability of suitable donors, the number of ARS victims that can be given an HSC transplant may be highly limited. Studies in animal models and clinical investigations suggest that the infusion of allogeneic MSCs alone may be an achievable and efficacious alternative strategy for stimulating hematopoietic recovery in patients [36,37]. A clinical approach that closely mimics this scenario is allogeneic MSC infusion to treat patients in which HSC engraftment has failed. In both cases, MSCs are administered without HSC coinfusion. MSC engraftment to the bone marrow enhances the HSC niche to a level that promotes the growth and maturation of residual HSCs. Although the currently available data are limited, MSC infusion as a rescue therapy for HSC graft failure has shown generally positive outcomes. Significant improvement in the bone marrow microenvironment and, in some cases, rapid and sustained hematopoietic recovery, have been observed in subjects who had failed to respond to conventional treatment [38-40]. To provide timely administration under this scenario, an option would be to stockpile allogeneic MSCs that would be readily available in the event of a radiological or nuclear emergency. This option is an attainable goal because relatively few donors would be required to generate a sufficient number of doses to treat hundreds to thousands of patients.

### Efficacy potential of MSC therapy for GI syndrome

In addition to hematological crisis, victims exposed to radiation levels exceeding 7 Gy will develop complications that are characteristic of radiation-induced GI syndrome. These symptoms include dehydration, electrolyte imbalance, malaise, anorexia, severe diarrhea, and fever. The basis of the injury to the GI tract is associated with the loss of rapidly dividing stem cells of the intestinal crypts, which replenish the epithelial layer of the gut during normal tissue turnover. The resulting breach in the GI barrier, in combination with immune suppression, presents a high risk of life-threatening infection. Patients diagnosed with GI syndrome have an extremely low survival rate, and no currently approved medical countermeasure specifically addresses this component of ARS.

Similar to the influence of MSCs on HSCs within the bone marrow compartment, both *in vitro* and *in vivo* data support the role of MSC therapy in promoting the restoration of intestinal epithelium by nurturing the growth of the residual surviving crypt stem cells. A mechanism through which this support occurs is the local secretion of growth factors and chemotactic signals that draw in epithelial progenitors to the sites of tissue damage. Preclinical models of abdominal and total body irradiation have demonstrated increased survival with MSC infusion accompanied by intestinal crypt cell regeneration, restitution of the stem cell niche, and increased xylose absorption. Serum levels of intestinal radioprotective factors (including R-Spondin1, KGF, PDGF and FGF2) and anti-inflammatory cytokines were increased, whereas inflammatory cytokines were down-regulated [41-43].

Clinical data specifically related to irradiation injury of the gut are extremely limited. However, systemic allogeneic MSC infusion to accidentally over-irradiated prostate cancer patients with radiation-induced colitis has been reported. These patients experienced a reduction in pain, diarrhea, hemorrhage, inflammation, and fistulization [44]. Additionally, successful clinical use of MSC therapy has been reported in treating certain inflammatory bowel diseases that involve the significant loss of intestinal epithelium, such as GvHD [45] and Crohn’s disease [46]. Considering the highly similar pathophysiology of these conditions, the same mechanisms that are active in the treatment of GvHD and Crohn’s disease may also be effective against the GI symptoms of ARS. A well-characterized model of GI syndrome in non-human primates recently developed by MacVittie et al. [47] may prove to be an invaluable tool for developing countermeasures for GI syndrome.

### Therapeutic rationale for MSC treatment of cutaneous and combined injury

Battlefield injuries commonly involve severe and extensive cutaneous tissue damage. The efficacy of MSC treatment in promoting skin regeneration has been shown in multiple preclinical injury models, including laceration [48], thermal burn [49], and radiation exposure [50-52]. The results of these studies demonstrate more expedient wound closure, decreased incidence of infection, increased vasculogenesis and elasticity, and reduced scar formation. Lacerative injury concurrent with irradiation presents a daunting treatment challenge. This scenario has been specifically evaluated by Hao et al. ([53]). A combined radiation–wound injury was generated in rats by producing an excisional wound equal to 2% body surface in subjects that had received 6 Gy total body irradiation. MSCs were then directly injected into the wound bed and margins. At 14 days post-injury, the wound area was approximately half the size in MSC-treated animals vs. control subjects.

Evidence that MSCs play a natural role in the process of skin regeneration in humans has been collected in a clinical study, in which the number of MSCs circulating in the peripheral blood of thermal-burn patients was quantified and compared to the number of circulating MSCs in the blood of healthy volunteers [54]. MSC phenotype was determined by the positive expression of five specific cell surface markers and the negative expression of eight other markers. The percentage of MSCs in circulating blood was more than 20-fold greater in burn patients compared to that in healthy individuals, and the degree of increase was correlated with the size and severity of the burn. These results offer data from human subjects that suggest MSCs play an important role in skin regenerative processes because the cells appear to be mobilized from the bone marrow in response to injury.

In addition to MSC efficacy in facilitating hematopoietic recovery and in promoting gut and skin repair, both structural and functional improvements for a variety of other injury types have been described. For example, the results of preclinical injury studies suggest effective MSC-based treatment of skeletal muscle [55], bone [56], tendon [57], lung [58], brain [59], heart [60], cornea [61,62], liver [63], and kidney [64]. The homing ability and adaptability of cellular therapy to conditions that are local to the injury site make this approach ideal for medical defense, particularly in cases involving more than one injury site or injury type.

### MSC production methodology at United States Army medical research institute of chemical defense

A logical next step in expanding the clinical use of MSC therapy is to develop efficient and consistently reliable methods for the production of pharmaceutical quantities of MSC product. The United States Army Medical Research Institute of Chemical Defense (USAMRICD) has developed production methods designed to facilitate the clinical transition of an MSC-based countermeasure, with the intention of actualizing the availability of this promising cellular therapeutic to health care providers responsible for treating warfighters [65]. The establishment of a defined MSC manufacturing paradigm will be critical for the progression of the technology to the Army’s advanced development group (United States Army Medical Materiel Development Activity, USAMMDA), for work leading to potential FDA approval. Although this report has focused on the development of MSC technology to produce a medical countermeasure to ARS, such a product may be significant in the treatment of additional battlefield indications (such as trauma, chemical exposure, and blast injury).

One improvement in MSC production methodology has proven to be the inclusion of fibronectin (Fn) as a surface coating for culture vessels. Fn is one of the most abundant extracellular matrix proteins in the bone marrow. The presence of Fn in MSC culture provides an environment that more closely mimics the endogenous MSC niche and promotes overall cell health and retention of stem cell differentiation potential. A second modification of standard practice that we have put in place is to actively prevent high cell density, including the initial (passage 0) MSC culture. Although the maintenance of lower cell density is fairly easy to achieve during later cell passages, no report has suggested the dispersion of cells from original colonies formed by the initial plated bone marrow as a significant consideration in MSC production strategy. This approach ensures that cells do not experience high density confluency for significant periods at any stage of the production process.

**Figure 1:**
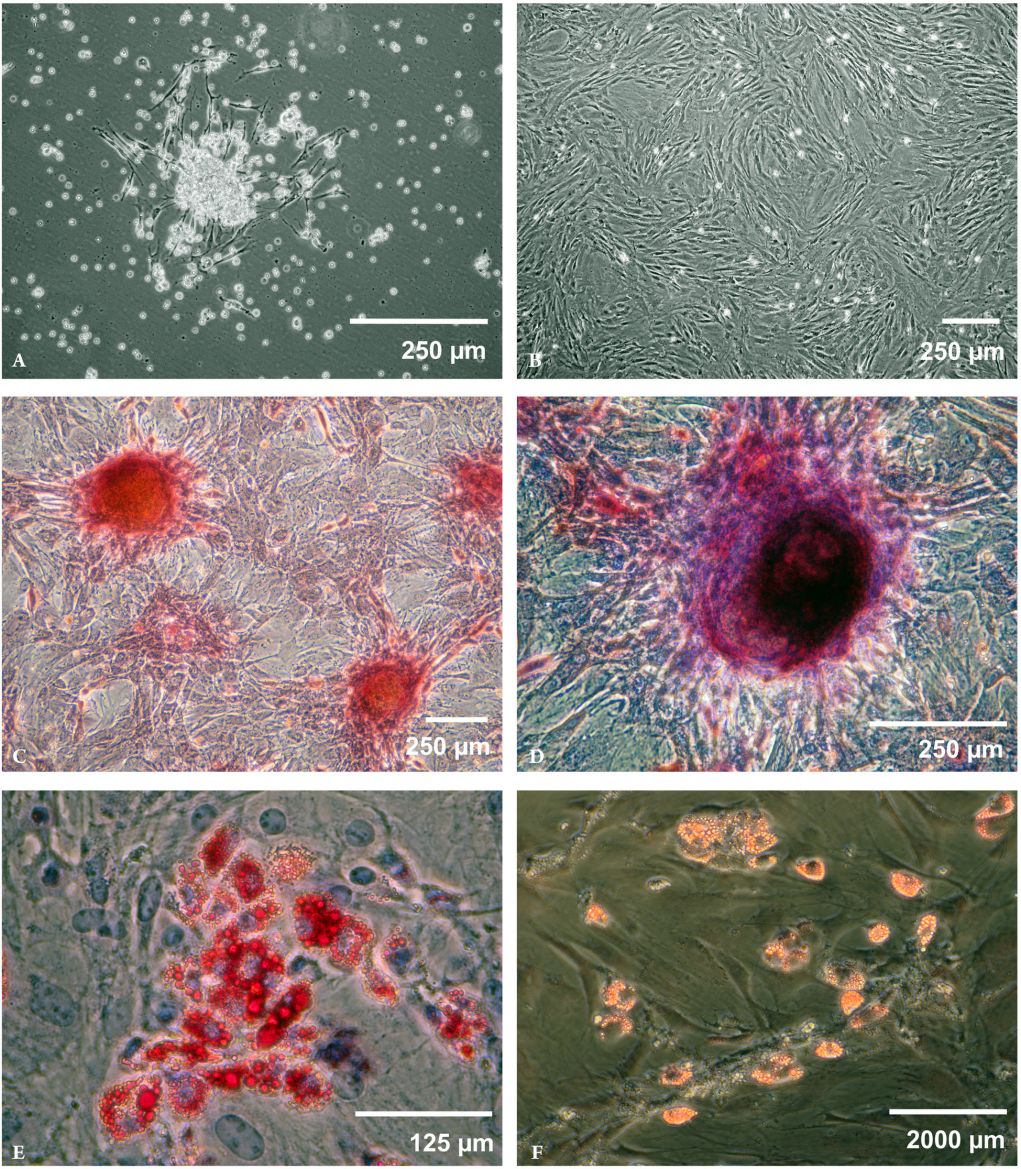
**Growth and differentiation of MSCs.** Freshly plated bone marrow contains mature blood cells, HSCs, and MSCs. Media changes result in the formation of small MSC colonies because of the differential adhesion properties of MSCs. These small groups of adherent cells generate an environment that supports the survival and growth of HSCs and **(Panel A)**. Long-term confluent MSC cultures form the typical swirled pattern characteristic of this cell type **(Panel B)**. MSCs can be induced to mature along the osteogenic differentiation pathway, as shown by calcium deposition staining **(Panel C)** and alkaline phosphatase activity staining **(Panel D)**. Adipogenic maturation is signified by Oil Red O staining **(Panel E)** and LipidTox Red uptake **(Panel F)**.

The methods we have established for MSC culture expansion have induced consistently high yields in production lots derived from human and animal bone marrow. Freshly plated bone marrow forms small MSC colonies that are seen to generate an environment that supports the survival and growth of HSCs (Figure 1A). Cultures left to grow to form a complete monolayer show the common swirled pattern that is typical of 100% confluent MSC cultures (B). Under appropriate conditions, MSCs can be induced to mature along the osteogenic (C-D) and adipogenic (E-F) pathways. Our methods allow for the generation of one production lot of high-quality, differentiation-capable MSCs that reach passage 3 in 16 days or less. “Passage 3” cells have completed an initial 5- to 7-day period of outgrowth from a plated bone marrow sample, followed by 3 rounds of expansion in cell culture that typically requires 2 to 3 days of growth each. In the absence of early passage 0 colony dispersal and an Fn-coated growth surface, MSC cultures require approximately twice the cultivation time to reach passage 3 [6,66,67]. A rapid MSC production methodology will be critical for applications that may require autologous MSC delivery, such as tissue engineering and reconstruction. Finally, the manufacturing process for MSC production has been designed to be completely scalable using commercially available “multi-stack” cultivation vessels, with final cell yields that solely depend on the volume of bone marrow input at culture initiation.

## Conclusion

The dynamic nature of medical threats that are faced by warfighters calls for the development of countermeasures that address a broad range of tissue pathologies. The adaptability of the cellular therapy approach discussed in this report provides a promising option to address the unmet needs that are critically important in medical defense. Current preclinical and clinical results of MSC efficacy investigations have demonstrated success in a variety of injury and disease settings that share a similar pathophysiology to the indications of interest.

The administration of MSCs by IV infusion has shown clinical benefit on surface wounds and can also reach tissue damage located deeper within the body. This capability is derived from the remarkable and well-demonstrated behavior of systemically delivered MSCs to distribute to sites of compromised tissue under the influence of chemokine homing signals produced at one or more injury sites. The injury-specific distribution of MSCs following infusion provides a particular advantage in the treatment of combined injury (e.g., laceration in combination with exposure to chemically or radiologically induced tissue damage). IV MSC delivery represents an approach in which the administration of a single therapeutic can facilitate tissue repair at multiple injury sites and for various injury types, in accordance with the specific requirements for the repair of each compromised site during the healing process. Significantly, the outstanding safety profile of this cellular therapeutic has been extensively demonstrated in the clinic. To date, more than 1,400 patients enrolled in clinical trials have received the first marketed MSC therapeutic, Prochymal® (Osiris Therapeutics, Inc.), with no reports of serious adverse events linked to treatment (ClinicalTrials.gov, September 2014 [3]).

The advancement of MSC therapeutics to address the broad array of rare but treatment-challenging injuries faced by warfighters will require the development of appropriate injury-specific preclinical models. Investigations designed to statistically evaluate MSC efficacy for the treatment of injuries induced by radiation, chemical, or blast exposure are critical for regulatory approval. The advancement of translational research directed toward treating battlefield injury will have multiple cross-over opportunities for dual-use applications within the general population. The challenging nature of modern warfare injuries requires that military medicine remain at the forefront of innovative medical approaches.

## Abbreviations

ARS: Acute radiation syndrome
MSC: Mesenchymal stem cell
Gy: Gray
GI: Gastrointestinal
GvHD: Graft vs. host disease
IV: Intravenous
MCP-1: Monocyte chemoattractant protein-1
MIP-1α: Macrophage inflammatory protein-1α
IL: Interleukin
SDF-1: Stromal derived factor-1
MHC: Major histocompatibility
VEGF: Vascular endothelial growth factor
HGF: Hepatocyte growth factor
IGF-1: Insulin-like growth factor-1
bFGF: Basic fibroblast growth factor
GM-CSF: Granulocyte-macrophage colony-stimulating factor
HSC: Hematopoietic stem cell
HLA: Human leukocyte antigen
USAMRICD: United States Army Medical Research Institute of Chemical Defense
USAMMDA: United States Army Medical Materiel Development Activity
Fn: Fibronectin
